# Metformin selectively targets redox control of complex I energy transduction

**DOI:** 10.1016/j.redox.2017.08.018

**Published:** 2017-08-26

**Authors:** Amy R. Cameron, Lisa Logie, Kashyap Patel, Stefan Erhardt, Sandra Bacon, Paul Middleton, Jean Harthill, Calum Forteath, Josh T. Coats, Calum Kerr, Heather Curry, Derek Stewart, Kei Sakamoto, Peter Repiščák, Martin J. Paterson, Ilmo Hassinen, Gordon McDougall, Graham Rena

**Affiliations:** a Division of Molecular and Clinical Medicine, Ninewells Hospital and Medical School, University of Dundee, Dundee DD1 9SY, Scotland, UK; b MRC Protein Phosphorylation and Ubiquitylation Unit, College of Life Sciences, University of Dundee, Dow Street, Dundee, Scotland, UK; c Institute of Chemical Sciences, School of Engineering & Physical Sciences, Heriot-Watt University, Edinburgh, EH14 4AS, Scotland, UK; d Environmental and Biochemical Sciences, The James Hutton Institute, Invergowrie, Dundee DD2 5DA, Scotland, UK; e Institute of Mechanical, Process and Energy Engineering, School of Engineering and Physical Sciences, Heriot-Watt University, Edinburgh, Scotland, UK; f Faculty of Biochemistry and Molecular Medicine, University of Oulu, Oulu, Finland

**Keywords:** Diabetes, Metformin, Mitochondria, NADH, NAD+

## Abstract

Many guanide-containing drugs are antihyperglycaemic but most exhibit toxicity, to the extent that only the biguanide metformin has enjoyed sustained clinical use. Here, we have isolated unique mitochondrial redox control properties of metformin that are likely to account for this difference. In primary hepatocytes and H4IIE hepatoma cells we found that antihyperglycaemic diguanides DG5-DG10 and the biguanide phenformin were up to 1000-fold more potent than metformin on cell signalling responses, gluconeogenic promoter expression and hepatocyte glucose production. Each drug inhibited cellular oxygen consumption similarly but there were marked differences in other respects. All diguanides and phenformin but not metformin inhibited NADH oxidation in submitochondrial particles, indicative of complex I inhibition, which also corresponded closely with dehydrogenase activity in living cells measured by WST-1. Consistent with these findings, in isolated mitochondria, DG8 but not metformin caused the NADH/NAD^+^ couple to become more reduced over time and mitochondrial deterioration ensued, suggesting direct inhibition of complex I and mitochondrial toxicity of DG8. In contrast, metformin exerted a selective oxidation of the mitochondrial NADH/NAD^+^ couple, without triggering mitochondrial deterioration. Together, our results suggest that metformin suppresses energy transduction by selectively inducing a state in complex I where redox and proton transfer domains are no longer efficiently coupled.

## 1 Introduction

**Fig. 1:**
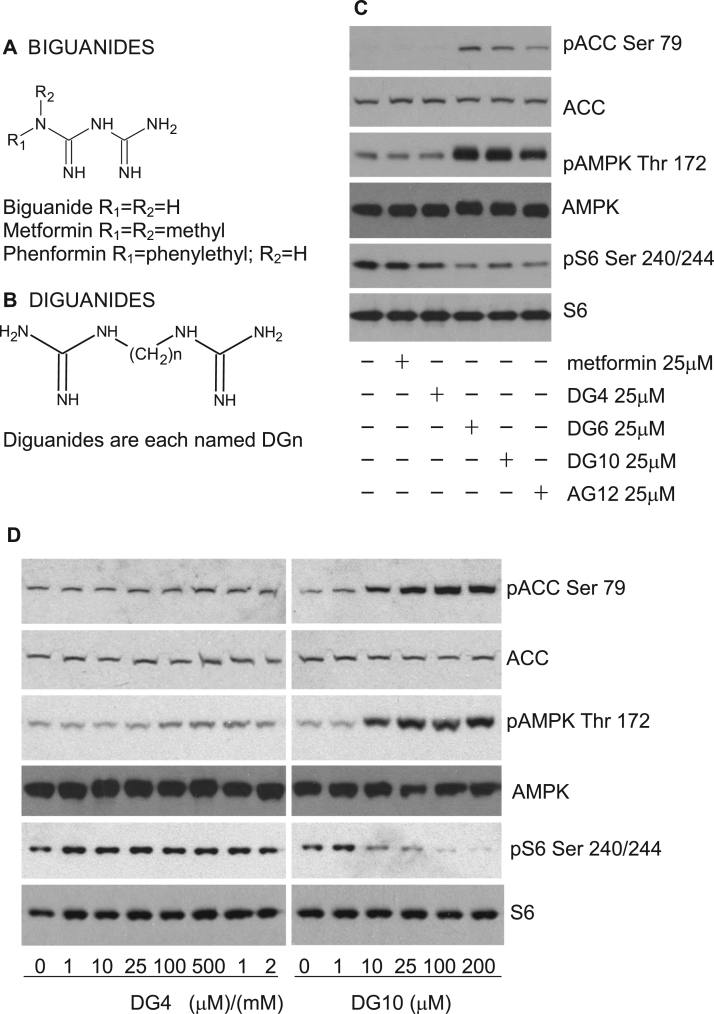
**Comparison of biguanide and diguanide compounds on cells.** Structures of biguanides *(a)* and diguanides *(b)* are shown. *(c, d)* H4IIE liver cells had serum removed for 2 h and then were treated with the compounds shown for 3 h, prior to lysis and SDS-PAGE as detailed in the Methods. Immunoblotting was performed using two ACC antibodies, one that detects ACC phosphorylated on Ser79 (pACC Ser 79) and another that detects ACC whether or not it is phosphorylated. A third antibody detects AMPK when it is phosphorylated on Thr 172 (pAMPK Thr 172) and a fourth detects AMPK whether or not it is phosphorylated. Finally two antibodies that detect S6 protein were used. One detects S6 phosphorylation of residues 240 and 244 (pS6 240/244), whilst a second detects S6 regardless of phosphorylation. For each section, the phosphorylation responses shown are representative of observations from at least three similar experiments. Quantitation of replicates by densitometry appears in the supplementary Fig. 1.

Recently we have been using analogues of metformin to study how cellular effects of metformin relate to its chemical properties, which include a strongly hydrophilic character, metal-binding and a pK_a_ within the physiological pH range [1], [2], [3], [4], [5], [6]. This collection of properties is not shared by other guanidine-containing compounds although many do have mitochondrial and antihyperglycaemic effects. In the 1920s for example, before the discovery of biguanides [7], [8], related compounds known as diguanides [9] were briefly evaluated as antidiabetic agents. Unlike biguanides, in diguanides, the two guanidine groups are separated by an alkyl chain of variable length (Fig. 1). In this series, antihyperglycaemic effects were found to increase, in proportion with toxicity, up to a linker size of 10–12 carbons [10], [11]. To achieve the maximum effect, the most potent and toxic diguanide, (*N*,*N*'-1,10-Decanediyl*bis*guanidine, synthalin A, termed DG10 in our study, Fig. 1b) was used in humans but caused liver toxicity [12]. Owing to this failure, there has been little investigation of the mechanism of action of synthalins. Although the mechanism of antihyperglycaemic effects is poorly understood for either drug class, inhibition of mitochondrial respiration has been observed in response to both diguanides and biguanides [13], [14]. Early studies suggested that mitochondrial effects might be due to the ability of guanidine compounds to alter the functional and physical structure of membranes [15], [16]; however, this model does not readily account for the widely differing toxicities and potencies of diguanides compared with biguanides and in addition metformin is one of the most hydrophilic biguanides, unlikely to interact significantly with membranes. Later studies suggested that metformin acts by inhibiting complex I in the electron transport chain [14], [17]. Mitochondrial suppression by metformin activates AMPK [18], [19], [20], which has emerged as a common cellular response to biguanides, thiazolidinediones and salicylates [19], [21], [22], [23]; however, there are other possible targets, some of which are AMPK-independent. These include regulation of glucose 6-phosphate levels [24], dephosphorylation of the ribosomal protein S6 by metformin [25], [26] and suppression of redox transfer by mitochondrial glycerophosphate dehydrogenase (mGPD) [27]. For mGPD, it has been argued elsewhere that effects on glucose production might require additional inhibition of the alternative redox malate/aspartate shuttle [28]. None of these models readily explain the suppression of weight gain that is one of the key clinical hallmarks of metformin treatment [29].

Recent work on complex I has found evidence for different modes of complex regulation by metformin compared with rotenone, which is a neurotoxic complex I inhibitor [30]. In the current study we have investigated differences between the actions of metformin, diguanides and other guanide containing drugs, as these differences might ultimately explain the lower toxicity of metformin compared with other electron transport chain inhibitors. Investigating the impact of these agents on cellular oxygen consumption, gluconeogenic gene regulation, signalling and glucose production, we found that phenformin and several diguanides induce effects similar to metformin on these outputs. Our studies have however isolated differences in mitochondrial responses to these other agents that are likely to contribute towards observed differences in toxicity.

## 2 Methods

### 2.1 Materials

Diamines used in synthesising diguanides, methylisourea and the compound AG12 were sourced from Sigma Aldrich. DG4 and DG10 came from Tocris. DG6 came from Santa Cruz Biotechnology. The antibodies used have been described previously [2], [23]. Briefly, the antibody to Ser79-phosphorylated acetyl-CoA carboxylase (ACC) was a gift from the Division of Signal Transduction Therapy at the University of Dundee. Total ACC, total AMP activated protein kinase (AMPK) α, Thr172-phosphorylated AMPKα, total S6 ribosomal protein and Ser 240/244-phosphorylated S6 antibodies for immunoblotting were from Cell Signalling Technology. Actin antibody was from Merck. Antibodies used in the AMPK activity assays were a generous gift from Prof D. Grahame Hardie at the University of Dundee. Chemical structures were drawn using ChemSketch.

### 2.2 Diguanide synthesis

Diguanides were synthesized using a method adapted from Tricot [31] by reacting 1 mol of a diamine with 2 mol of methylisourea. The methylisourea was dissolved in ice-cold ultrapure water and the pH increased to 10 by drop-wise addition of 5 M KOH. The diamine was added and this was incubated for three hours, performing 15 min checks to ensure that the pH was maintained at 10. The guanidinylated products are insoluble at pH 10 and precipitates formed were collected by centrifugation and washed twice with 10 ml ice cold ultrapure water by re-centrifugation. The final precipitates collected were frozen at −20 °C and freeze dried. The success of the syntheses was initially assessed by positive mode direct injection mass spectrometry, then the purity of the diguanides confirmed by liquid chromatography mass spectrometry with MS^2^ fragmentation using an ion-pairing separation method (see Supplementary materials for details). In our experience with this method, the purity of the product was dictated predominantly by the purity of the starting material.

#### 2.2.1 H4IIE cell culture and lysis for immunoblotting

Maintenance of H4IIE cells in DMEM (31885 Invitrogen) plus 5% Fetal calf serum (Seralab) has been described previously [2], [32], [33], [34]. Cells were used for no more than 30 passages. Briefly, prior to an experiment being carried out, fresh medium was added the evening before. Two hours prior to stimulation on the day of the experiment, cells were placed in DMEM without serum. Cells were scraped into ice-cold lysis buffer: (50 mM Tris acetate pH7.5, 1% (w/v) Triton ×100, 1 mM EDTA, 1 mM EGTA, 0.27 M sucrose, 50 mM NaF, 1 mM sodium orthovanadate, 10 mM β glycerophosphate, 5 mM sodium pyrophosphate, 1 mM benzamidine, 0.2 mM phenylmethylsulfonyl fluoride (PMSF) and 0.1% (v/v) β-mercaptoethanol) and then prepared for SDS-PAGE and immunoblotting as described previously [35], [36]. Protein concentration was measured using Bradford reagent (Pierce). Immunoblot densitometry was performed with Image Studio Lite version 5.2 (LI-COR).

#### 2.2.2 Preparation of cell extracts, immunoprecipitation and assay of AMPK

This was carried out essentially as described previously [2]. Briefly, cells were washed twice in ice-cold PBS then harvested in ice-cold lysis buffer (50 mM Tris-HCl, pH 7.4, 50 mM sodium fluoride, 5 mM sodium pyrophosphate, 1 mM EDTA, 1 mM EGTA, 150 mM sodium chloride, 1 mM dithiothreitol (DTT), 0.1 mM benzamidine, 0.1 mM PMSF, 1% Triton X-100, and 5 μg/ml soybean trypsin inhibitor). Lysates were cleared of debris by centrifugation at 13,000*g* for 15 min at 4 °C, and the protein concentration measured as in the previous section. Cell extracts were incubated overnight with protein G sepharose conjugated to both anti-AMPKα1 and AMPKα2 antibodies (generously gifted by Professor Grahame Hardie) [37]. Immunoprecipitates were pelleted and rinsed twice with 1 ml ice-cold buffer (as above but with 0.5 M NaCl) and once with ice-cold HEPES buffer (50 mM HEPES pH 7.4, 0.03% Brij-35 and 1 mM DTT). AMPK kinase activity was assayed at 30 °C, in the presence of 0.1 μCi of [γ-^32^P]ATP, 0.33 mM cold ATP, 8.3 mM MgCl_2_, 0.33 mM AMP, and 0.33 mM HMRSAMSGLHLVKRR (SAMS) peptide. Kinase activity is expressed as phosphate incorporation into 1 nmol substrate in 1 min per mg of protein. Each bar of a graph consists of data from at least six separate immunoprecipitations, each from a separate dish of cells.

### 2.3 Hydrophobicity

Molecular representations of log P were generated in Marvin Sketch, as were calculations of log P, with 1:1:1 weighting for VG [38], KLOP [39] and PHYSPROP approaches.

#### 2.3.1 Luciferase reporter and WST-1 assays

The reporter cell line LLHG stably expresses the human glucose 6-phosphatase promoter upstream of the luciferase reporter gene. This cell line was a generous gift from Dr Calum Sutherland. LLHG cells were plated into 12 well plates and left overnight. Cells were washed once in serum-free DMEM before serum starving for 6 h and subsequent overnight treatment with dexamethasone and cAMP [23] and drugs as indicated in the figures. For lysis, cells were washed once in PBS before addition of 1× Cell Culture Lysis Buffer (Promega). Wells were scraped and lysates spun for 2 mins, 4 °C, 13,000 rpm. For luciferase assay, 10 μl lysate was added to a 96-well, white walled, clear bottom plate and 100 μl luciferase assay reagent (Promega) added before quantification of luminescence. To correct for well-to-well variation, protein concentration was determined by Bradford assay. Data is represented as RLU/mg protein. Effects of each dose were measured at least six times, each from a separate well of cells. For WST-1 assays, H4IIE cells were seeded on a 96 well plate, placed in serum-free medium and treated with the appropriate drugs. WST-1 mixture (Roche) was added to each well, mixed and incubated for two hours. Effects of each dose were measured at least eight times, each from a separate well of cells. The absorbance of each sample was measured using a spectrophotometer at a wavelength of 440 nm.

#### 2.3.2 Measurement of whole-cell oxygen consumption rate

Using the Seahorse XF24, OCR was measured as described previously [2]. Briefly, cells were plated at a density of 1 × 10^6^ cells per well in serum-containing medium. Once attached, cells were washed once in serum-free DMEM and incubated for 2 h with de-gassing for the final hour. For the assay, cells were incubated in unbuffered DMEM containing 1 g/L glucose, 200 mM Glutamax-1, 100 mM sodium Pyruvate, 30 mM NaCl. Baseline OCR was measured for 30 mins before injection of appropriate compounds. OCR was continuously measured for a period of 145 min before uncoupling by addition of 100 uM 2,4 dinitrophenol (2,4, DNP).

### 2.4 Glucose assay

Treatment of cells for hepatocyte glucose production was carried out essentially as described previously, using primary mouse hepatocytes [2], [5], [23], [40]. Glucose production was determined after a 12 h incubation period in glucose-free DMEM with or without diguanide analogues, 2 mM metformin or 100 µM cAMP (Calbiochem, 116812). At the end of the incubation period of 12 h, medium was collected and glucose concentration determined by the hexokinase/glucose-6-P dehydrogenase method. Each bar of a graph consists of data from at least three separate measurements, each from a separate dish of cells, assayed by GAGO assay as described earlier [5].

#### 2.4.1 Submitochondrial particles and intact mitochondria

Rat liver mitochondria were prepared in 70 mM sucrose, 210 mM mannitol, 5 mM HEPES, 1 mM EGTA and 0.5% fatty-free bovine serum albumin, pH 7.2 essentially as described by [41], [42]. The excised liver was cut with scissors to approx. 4-mm diameter pieces and homogenized in a Teflon-pestle Potter-Elvehjem homogenizer with two passes at 300 rpm. The homogenate was centrifuged at 750×*g* and the supernatant centrifuged at 8000×*g*. The pellet was resuspended in the isolation medium and centrifuged at 8000×*g* and the pellet resuspended in a volume of 1 ml/g original liver weight and kept in an ice bath.

Submitochondrial particles (SMP) were prepared according to [43] by suspending rat liver mitochondria in 30 mM potassium phosphate buffer, 0.4 mM EDTA and sonication for a total of 120 s with a 50% duty cycle in a Branson Sonifier Cell Disruptor B-30 using power setting 4 and a micro tip. The suspension was centrifuged at 8000×*g* and the resulting supernatant at 105,000×*g* for 45 min in a Ti60 fixed-angle rotor in a Beckman L8-70 M ultracentrifuge. The pellet was suspended in 30 mM potassium phosphate, pH 7.4. All manipulations, including chopping the liver, homogenizing, re-suspending and sonication were performed on an ice/water bath, centrifugation, and ultracentrifugation were performed in refrigerated centrifuges in rotors pre-cooled to 4 °C. Protein concentration in mitochondria and submitochondrial particles was measured with the biuret method.

##### 2.4.1.1 Fluorometry

NADH (excitation 340 nm, emission 460 nm) fluorescence was measured in a Fluoromax-2 spectrofluorometer (Horiba Jobin Yvon, Inc., Edison, NJ, U.S.A).

Oxygen consumption by mitochondrial suspensions was measured with a polarographic Rank Brothers oxygen electrode with Perspex chamber (Rank Brothers, Cambridge, U.K.) with a magnetic stirrer. In experiments where fluorescence and oxygen consumption were monitored simultaneously, the fluorometric 1 cm × 1 cm cuvette was equipped with a magnetic stirrer, a stopper with an injection port and a miniature oxygen electrode (MI-730, Microelectrodes, Inc., Bedford, NH, U.S.A.).

#### 2.4.2 Complex I electron transfer to hydrophilic electron acceptors

This was measured using the MitoTox Complex I OXPHOS Activity Microplate Assay (AbCam, ab109903). The assay was performed essentially following the manufacturer's instructions. The only difference was that ubiquinone was substituted with either 200 µM paraquat (Sigma, 36541) or 1 mM K_3_Fe(CN)_6_ (Sigma, 702587). Differing doses of metformin and DG5 as indicated were used as the compounds to be tested.

### 2.5 Data and statistical analyses

Errors in bar graphs are expressed as mean ± SEM. Comparisons between groups were made by one-way or two-way ANOVA with post-hoc test using Prism. EC50s and IC50s were determined using Prism. Differences were considered statistically significant if *P* was less than 0.05. *** denotes p < 0.001; ** denotes p < 0.01 and * denotes p < 0.05.

## 3 Results

### 3.1 Diguanides induce metformin-like effects on AMPK signalling and S6 phosphorylation

In the first experiments we compared the effect of commercially available diguanides with metformin (Fig. 1a,b) on AMPK and S6 signalling, focusing on metabolic signalling responses that we have studied previously in liver cells [2], [5]. At a single concentration, we found that two diguanides, 1,10-Bis(guanidino)decane (synthalin A, termed DG10 in this study) and 1,6-Bis(guanidino)hexane (DG6 in this study) had effects on both AMPK and S6 phosphorylation at 25 μM, whereas 1,4-Bis(guanidino)butane (arcaine, DG4 in this study) was ineffective (Fig. 1c, quantified in supplementary Fig. 1). Previous studies also established that alkylguanidines with chain lengths comparable with synthalin act as even more potent mitochondrial inhibitors than diguanides or biguanides [13] and therefore we tested dodecylguanidine (AG12), the most potent of these, and found that it too activated AMPK and suppressed S6 phosphorylation (Fig. 1c), demonstrating that the second guanidine group is dispensable for effects of diguanides on these signalling pathways.

**Fig. 2:**
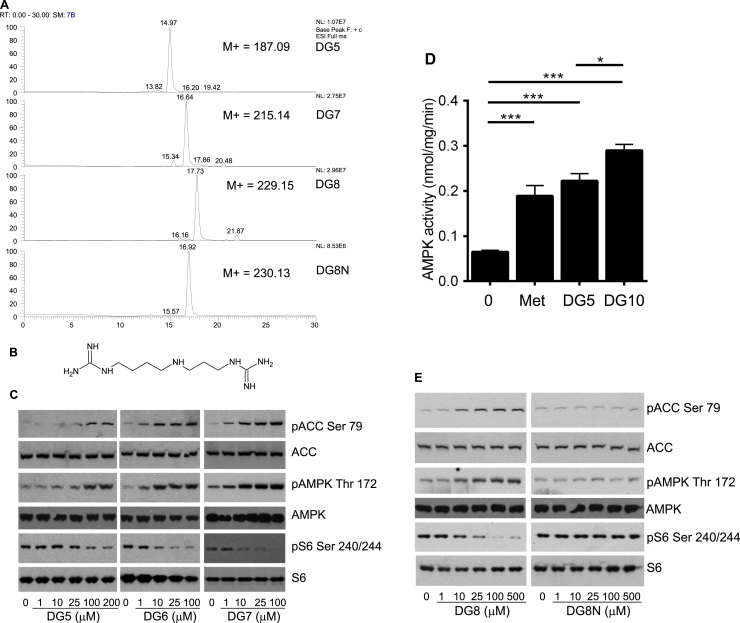
**Synthesis and analysis of additional DG compounds and effects on cells.***(a)* LCMS of synthesized diguanide compounds. *(b)* DG8N structure. Hydrophobicity maps are presented in supplementary Fig. 3. *(c,e)* H4IIE liver cells had serum removed for 2 h and then were treated without (‘0’) or with the compounds shown for 3 h, prior to lysis and SDS-PAGE as detailed in the Methods. The dose responses shown are representative of observations from at least three similar experiments, as detailed in Table 1a. Densitometric determination of IC50s and EC50s of replicates is also given in Table 1a. AMPK activity in response to 2 mM metformin, 100 μM DG5 and 10 μM DG10 treatment is detailed in *(d)* Assay of AMPK immunoprecipitated from drug-treated cells. A one-way ANOVA was carried out to determine differences between treated and untreated columns. The p value comparing DG5 and DG10 is also shown. Each bar of the graph is composed of data derived from at least eight separate immunoprecipitations.

In dose response experiments, we found that DG4 induced no apparent changes in AMPK, ACC or S6 at 2 mM, whilst the effects of DG10 on these readouts were apparent in the low micromolar range (Fig. 1d, calculated EC50s on ACC and AMPK and IC50s on S6 phosphorylation in Table 1a). Previous studies on glycaemia carried out in the 1930s [44] also found that diguanides with more than four carbons act very much more potently than biguanides. To study this further, we synthesized diguanides that are not commercially available, DG5, DG7 and DG8, using a modification of an earlier synthesis protocol. In this reaction, the products precipitate but the reactants remain in solution, which enabled us to obtain very pure preparations. Indeed, LC-MS analysis yielded single peaks of the expected mass for all diguanide compounds with estimated purity >95% (Fig. 2a). MS and MS^2^ data are presented (Supplementary Fig. 2). One effect of increasing chain length is increased hydrophobicity, and to enable a further test of the importance of this property in the action of the diguanides, an additional analogue was synthesized where the alkane chain in DG8 was disrupted by replacement with an amine group, to generate DG8N (Fig. 2b). Calculated hydrophobicity maps are presented showing the effects of this substitution compared with DG8 (Supplementary Fig. 3a,b). DG8N has a lower hydrophobicity than DG4 (clogD −10.08 against −7.68).

**Table 1:** Densitometric determination of EC50s (μM) and 95% CI of diguanides on ACC and AMPK phosphorylation and IC50s on S6 phosphorylation.

A	DG4	DG5	DG6	DG7	DG8	DG10	DG8N
pACC	>500 n = 3	11.0 (5.2–23.3) n = 3	2.4 (1.3–4.6) n = 4	3.4 (1.8–6.2) n = 5	3.1 (1.3–7.6) n = 3	2.2 (1.0–4.9) n = 4	>500 n = 3
pAMPK	>500 n = 2	12.9 (4.7–35.1) n = 3	1.4 (0.6–3.0) n = 3	3.7 (1.6–8.4) n = 4	2.5 (1.5–4.2) n = 3	1.2 (0.8–1.8) n = 4	>500 n = 3
pS6	>500 n = 3	536 (163–1750) n = 3	2.8 (1.8–4.1) n = 3	4.9 (2.3–10.3) n = 5	14.8 (11–19) n = 3	3.9 (2.0–7.3) n = 4	>500 n = 3

B	2-methyl DG6	1-ethyl	2,2-dimethyl
		DG5	DG5
pACC	16 (7.4–33) n = 3	>200 n = 3	>200 n = 3
pAMPK	27 (18–40) n = 3	>200 n = 3	>200 n = 3
pS6	32 (24–43) n = 3	>200 n = 3	>200 n = 3

### 3.2 A hydrophobicity threshold affecting potency of diguanides on AMPK, gluconeogenic gene expression and hepatocyte glucose output

We examined effects of synthesized diguanides on cell signalling (Fig. 2c) and calculated EC50s for ACC/AMPK phosphorylation and IC50s for S6 phosphorylation by analysis of densitometry data from blots. We found that DG5 was less potent than DG7, DG8 and DG10 on ACC, AMPK and S6 phosphorylation, with EC50s and IC50s for these latter drugs in the low micromolar range (Table 1a). The lack of effect of DG4 suggests that a chain length threshold of five is required for effects at this time-point. When we assayed effects of the drugs on cell AMPK activity, we found that DG5, the least potent of the active compounds, and DG10, the most potent, both increased AMPK activity at least as well as metformin and at much lower concentrations (Fig. 2d). To investigate further whether either chain length or hydrophobicity correlated with the potency of diguanide analogues, we compared DG8 with DG8N. In parallel experiments, we demonstrated low micromolar efficacy of DG8 on signalling responses (Fig. 2e) whilst in contrast, DG8N was inactive at much higher concentrations (Fig. 2e).

**Fig. 3:**
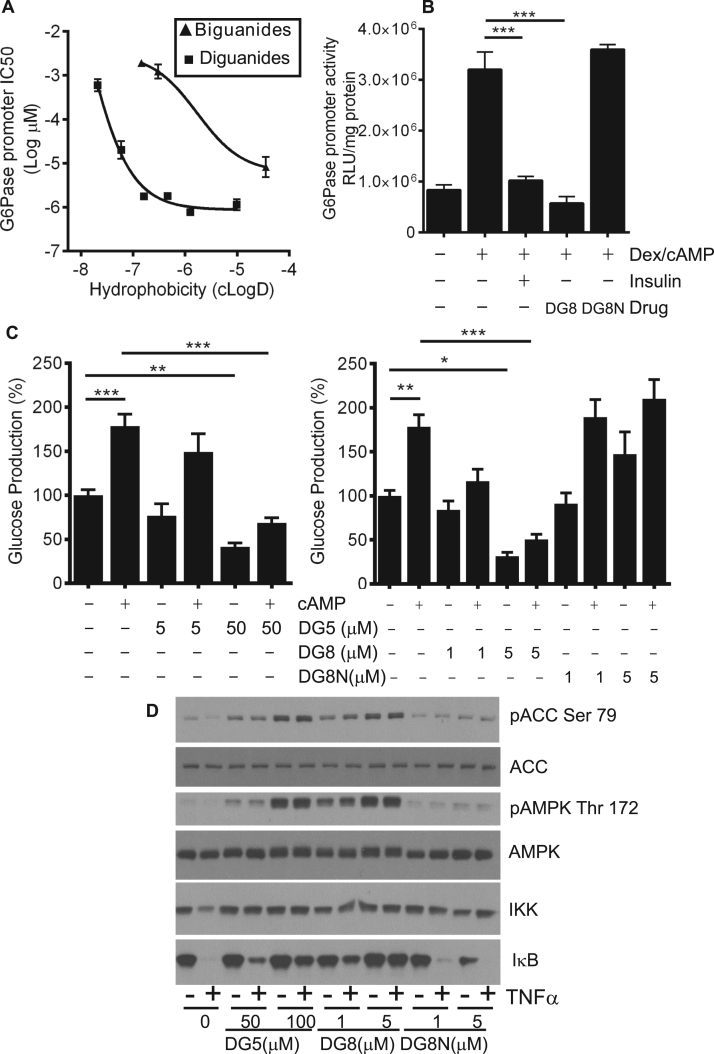
**Relationship between EC50 on G6Pase promoter activity, hepatocyte glucose production and hydrophobicity of the drugs.***(a)* IC50s were determined for each drug on G6Pase promoter activity using LLHG cells. Calculated hydrophobicity for each compound was also determined. The relationship between log IC50 and clogD is presented. Plot of (left to right) biguanide, metformin and phenformin **(triangles)** and (left to right) DG4, DG5, DG6, DG7, DG8 and DG10 **(squares)**. A lower log IC50 indicates a more potent compound, these values are tabulated in Table 2. Hydrophobicity plots of selected structures are provided in supplementary Figs. 3 and 4. *(b)* Comparison of 1 μM DG8 and 1 mM DG8N on G6Pase promoter activity. Dex/cAMP was used to stimulate the promoter. Ins denotes insulin. In (a) and (b) effects of each dose were measured at least six times, each from a separate well of cells. *(c)* Primary hepatocytes were treated in the presence or absence of DG5, DG8 and DG8N with and without 100 µM cAMP and glucose production measured as described in the Methods. A one-way ANOVA was carried out. *(d)* Primary hepatocytes were serum starved overnight and then were treated with the diguanides shown for 3 h, prior to 15 min treatment with TNFα before lysis and SDS-PAGE as detailed in the Methods. Immunoblotting was performed using antibodies described in Fig. 1. In addition, data is shown from an antibody for IKKα and one for IκB. The phosphorylation and protein degradation responses for IκB are representative of observations from at least three similar experiments. Quantitation of these replicates by densitometry appears in Supplementary Fig. 5.

The western blotting data outlined above suggests that diguanides DG4-6 are progressively more potent as hydrophobicity increases, plateauing from DG6 upwards (Fig. 1, Fig. 2, e). We recognize that western blotting is best described as a semi-quantitative technique and to carry out further comparison of the diguanides and biguanides, we measured metformin-like effects of the compounds on activity of the glucose 6-phosphatase promoter (Table 2). Plotted on a graph, potency of the diguanides and biguanides both increased with the calculated LogD, with a sharp potency step-up, greatly lowering IC50s between DG4 and DG6 in diguanides and between biguanide/ metformin and phenformin in the biguanide drugs (Fig. 3a, squares L-R are DG4, DG5, DG6, DG7, DG8, DG10, whilst triangles L-R are biguanide, metformin, phenformin). The difference in hydrophobicity between metformin and phenformin due to the presence of the phenyl ring in phenformin is presented in Supplementary Fig. 4a,b. The IC50 of phenformin is well within the range exhibited by DGs, whilst the other two biguanides are less potent (Fig. 3a). A comparison of DG8 and DG8N showed a striking difference between the potencies of these two analogues, as DG8 inhibited the gene promoter at concentrations as low as 1 μM, while in contrast DG8N was ineffective at millimolar levels (Fig. 3b).

**Table 2:** IC50s (μM, except values in italics, mM) and 95% CI of drugs on G6Pase luciferase promoter activity and IC50s determined by WST-1 assay.

	DG4	DG5	DG6	DG7	DG8	DG10	Biguanide	Metformin	Phenformin
G6Pase	597 (435–820)	20.0 (12.6–31.8)	1.8 (1.6–2.0)	1.8 (1.5–2.1)	0.8 (0.7–0.9)	1.1 (0.9–1.5)	*1.9 mM (1.6–2.3)*	*1.2 mM (0.8–1.8)*	8.2 (4.8–13.9)
WST-1	173.5 (124.2–242.3)	39.0 (10.9–138.9)	4.5 (3.2–6.4)	4.0 (2.8–5.5)	1.6 (1.2–2.0)	3.4 (2.3–5.1)	>500	>500	57.8 (26.1–127.6)

We also investigated the effects of DG5, DG8 and DG8N on hepatocyte glucose production, as this is thought to be the major clinical hallmark of metformin action [45], [46]. We found that the effects of the drugs were similar to those that we had observed on AMPK phosphorylation, as DG5 and DG8 both suppressed hepatocyte glucose production, in a dose-dependent manner whilst DG8N was ineffective (Fig. 3c). In primary hepatocytes we found a similar pattern of signalling, with DG5 and DG8 active but DG8N inactive on AMPK and ACC phosphorylation (Fig. 3d). In addition, consistent with recent work of ours that mitochondrial inhibition by biguanides can inhibit NF-κB signalling [5], we found that DG5 and DG8 but not DG8N inhibited TNFα-induced IκB degradation (Fig. 3d and Supplementary Fig. 5a-c).

### 3.3 A minimum hydrocarbon chain length affects potency of diguanides on AMPK, gluconeogenic gene expression and hepatocyte glucose output

**Fig. 4:**
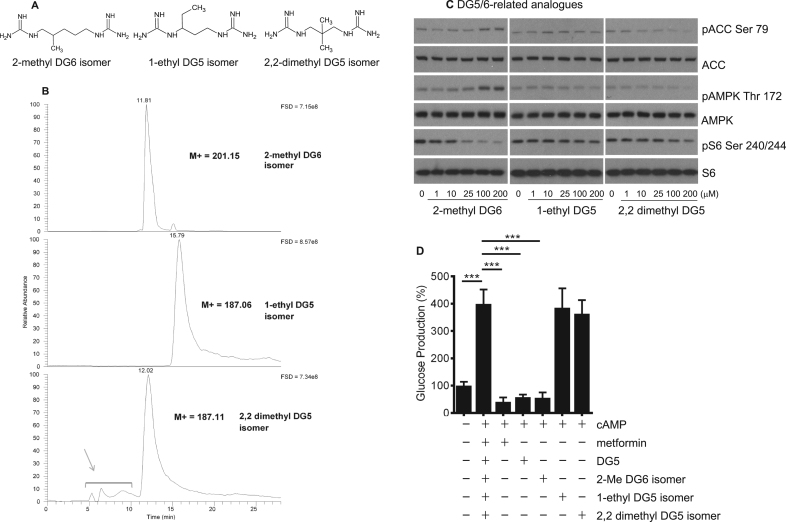
**Cell effects of branched diguanide isomers.***(a)* Structures of diguanide isomers. *(b)* LCMS of synthesized compounds. Grey arrow denotes guanidylated impurities carried over from starting material. *(c)* H4IIE liver cells had serum removed for 2 h and then were treated with the compounds shown for 3 h, prior to lysis, SDS-PAGE and immunoblotting as detailed in the Methods and Fig. 1. Densitometric determination of IC50s and EC50s of these replicates is also given in Table 1a, c, n=3. *(d)* Primary hepatocytes were treated in the presence or absence of 2 mM metformin (Met), 50 µM DG5, 100 µM 2-methyl DG6 isomer, 100 µM 1-ethyl DG5 isomer or 100 µM 2,2 dimethyl DG5 isomer and glucose production measured as described in the Methods. Each bar of a graph consists of data from at least three separate measurements, each from a separate dish of cells. A one-way ANOVA was carried out.

Next, we examined the effect of branching of the hydrocarbon chain connecting the guanides in the DG molecule. We synthesized three further molecules, depicted in Fig. 4a. 2-methyl DG6 is a branched isomer of DG6. In this molecule the length of the hydrocarbon linker remains the same as the threshold compound DG5. In addition, two branched isomers of DG5 were generated with shorter hydrocarbon linkers. LCMS data in Fig. 4b indicated each compound was >95% pure, with the exception of the 2,2-dimethyl isomer with estimated purity of 90%. MS and MS^2^ data is presented in Supplementary Fig. 6a,b. When we compared these analogues side by side, we found that only the 2-methyl DG6 isomer induced AMPK signalling and suppressed S6 phosphorylation (Fig. 4c, Table 1b) and hepatocyte glucose production (Fig. 4d), indicating that in addition to possible contributions of hydrophobicity in itself, the DG compounds’ low micromolar signalling effects depend on a minimum hydrocarbon chain length of 5 carbons.

### 3.4 Mitochondrial responses to phenformin and diguanides differ from metformin

**Fig. 5:**
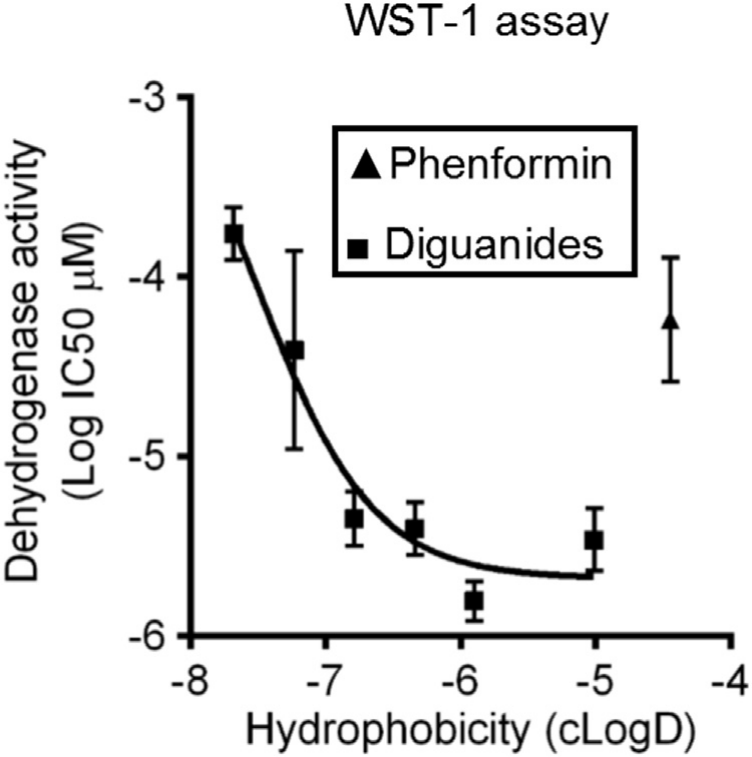
**Relationship between inhibition of dehydrogenase activity in cells (IC50) and hydrophobicity of the drugs.** IC50s on cellular dehydrogenase activity were determined for each drug using WST-1, tabulated in Table 2. Calculated hydrophobicity for each compound was used as for Fig. 3a to depict the relationship between log IC50 and clogD. Plot of phenformin **(triangle)** and (left to right) DG4, DG5, DG6, DG7, DG8 and DG10 **(squares)**. A lower log IC50 indicates a more potent compound. Effects of each dose were measured at least six times, each from a separate well of cells.

**Fig. 6:**
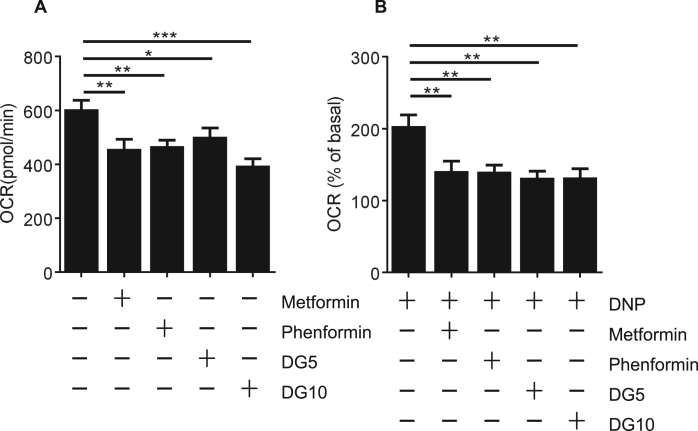
**Mitochondrial responses to biguanides and diguanides.** H4IIE cells were treated with the drugs shown (2 mM metformin, 250 μM phenformin, 100 μM DG5 and 10 μM DG10) and oxygen consumption measured in a Seahorse metabolic flux analyzer. Baseline OCR was measured for 30 mins before injection of appropriate compounds. OCR was continuously measured for a period of 145 min (a) before uncoupling by addition of 100 μM 2,4 dinitrophenol (2,4, DNP) for 10 min (b). A one-way ANOVA was carried out. Each data point consist of measurements from at least eight separate wells of cells.

**Fig. 7:**
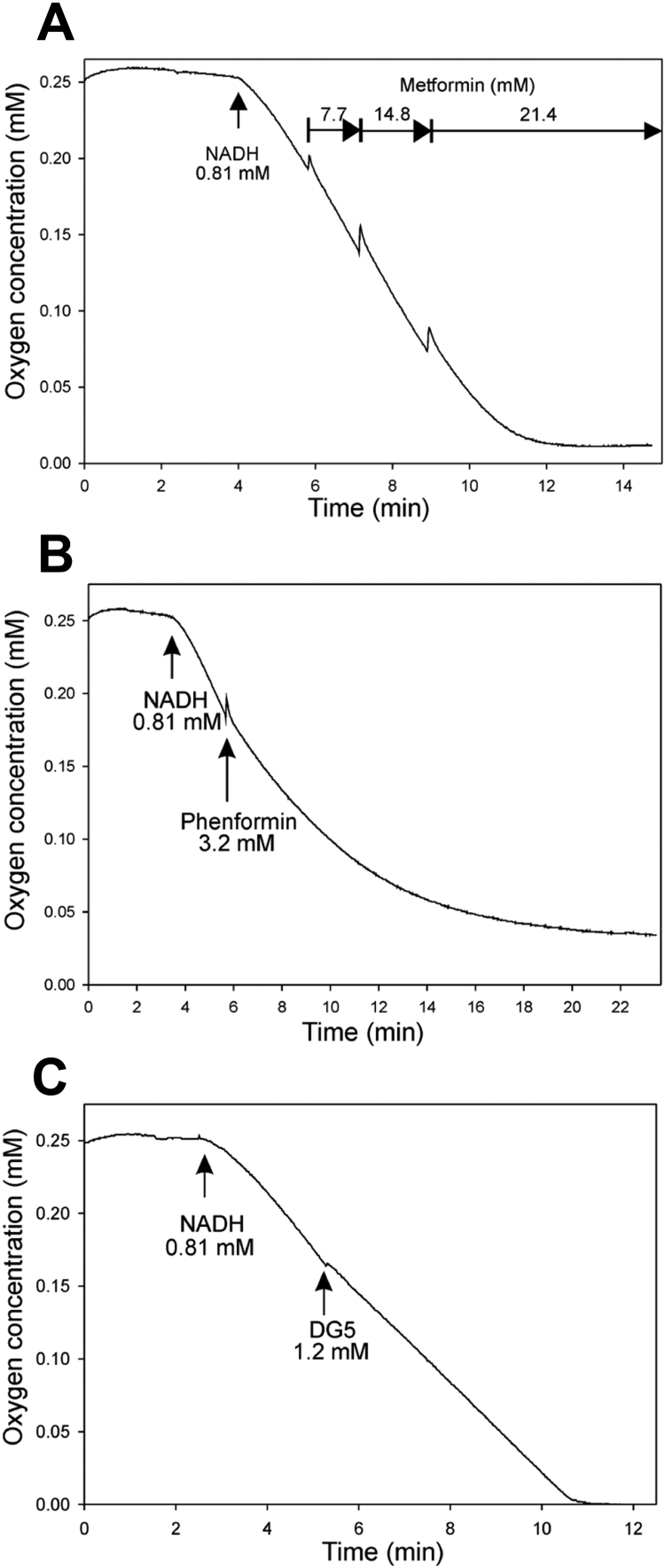
**Effects of biguanides and diguanides on NADH oxidation in submitochondrial particles from rat liver.** Representative responses to metformin n=5 *(a)*, phenformin n=4 *(b)* and DG5 n=3 *(c)* on NADH oxidation in SMPs. Incubation conditions: 137 mM KCl, 19.5 mM HEPES, 5.6 mM potassium phosphate, 2.9 mM MgCl_2_, 1 mM EDTA, pH 7.2. 0.23 mg protein, total volume 0.615 ml.

**Fig. 8:**
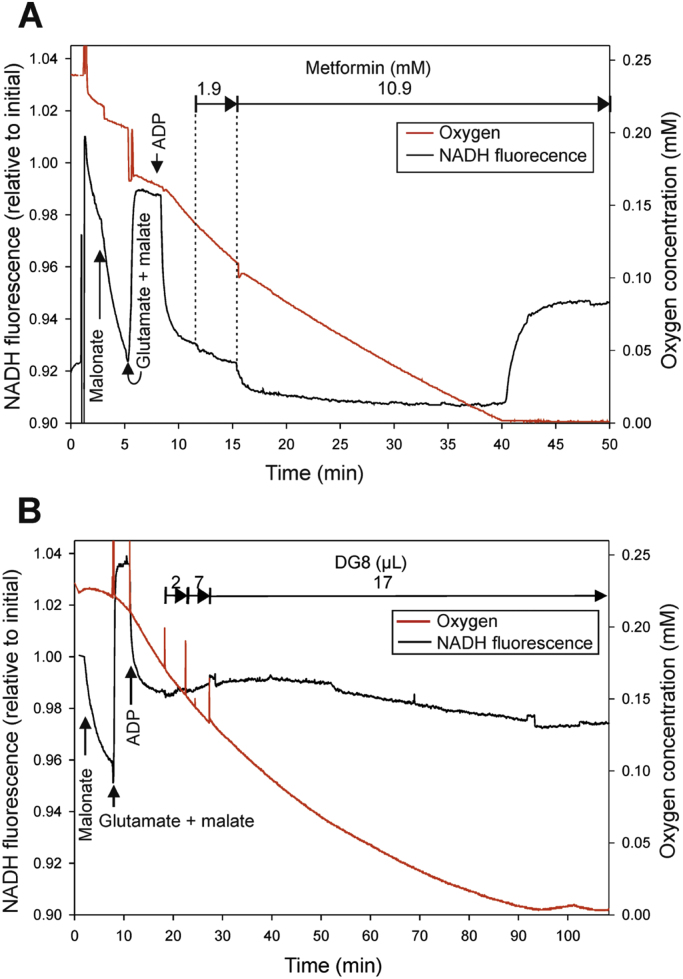
**Effects of metformin and DG8 on NADH fluorescence in isolated rat liver mitochondria.** Incubation conditions 130 mM KCl, 2.9 mM mannitol, 3 mM sucrose, 19 mM HEPES, 4.6 mM potassium phosphate, 2.8 mM MgCl_2_, 0.9 mM EDTA, 0.04 mM EGTA, 0.02% fatty acid-free bovine serum albumin. 1.7 mg protein, total volume 2.6 ml, pH 7.2. Representative responses are presented, each drug was tested at least three times. *(a)* Effect of metformin on NADH fluorescence and oxygen consumption. Final concentrations after addition as marked in the graph: 2.7 mM malonate 5.3 mM glutamate, 2.7 mM malate, 1 mM ADP, metformin 1.9 mM, metformin 10.9 mM. *(b)* Effect of DG8 on NADH fluorescence and oxygen consumption. Final concentrations after additions: 2.7 mM malonate 5.3 mM glutamate, 2.7 mM malate, 1 mM ADP. Aqueous saturated solution of DG8 was added three times as indicated.

One possible explanation for the overlap in potencies that we observed between phenformin and diguanides is that there are targets for hydrophobic guanide-containing compounds that are inhibited by phenformin and with progressively increasing potency by diguanides. To study this further we investigated whether other cellular effects of phenformin correlate more closely with diguanides than metformin. Consistent with this we found that phenformin and diguanides inhibited mitochondrial dehydrogenase activity in cells, with IC50s in the WST-1 assay corresponding very closely with IC50s on the G6Pase promoter (Table 2, Fig. 5. DGs left to right are DG4, DG5, DG6, DG7, DG8 and DG10. Phenformin is the only triangle), whilst in contrast, we were unable to establish IC50s in the WST-1 assay for metformin and biguanide, despite them efficiently inhibiting G6Pase (Fig. 3a). In Seahorse microplate studies to measure mitochondrial respiratory activity in cells, we found that DG5, DG10, metformin and phenformin each suppressed oxygen consumption to a similar degree (Fig. 6a,b). In other experimental approaches however, further marked differences were detected between metformin and the other drugs. In a conventional oxygen electrode chamber, direct measurement of effects on oxidation of the NADH/NAD^+^ couple by liver submitochondrial particles corresponded much more closely with effects on the WST-1 assay. Effects of both phenformin and DG5 on NADH oxidation were observed at much lower concentrations than responses to metformin (Fig. 7). The effects of metformin and DG8 on intact mitochondria were also different. Treatment of mitochondria with DG8 modestly reduced oxygen consumption and reduced the NADH/NAD^+^ couple, consistent with complex I inhibition (Fig. 8). Once oxygen was depleted however, the NADH/NAD^+^ couple did not respond, indicative of mitochondrial deterioration. Metformin treatment also modestly reduced oxygen consumption but in contrast to DG8, this was accompanied by acute oxidation of the NADH/NAD^+^ couple (Fig. 8) and in addition, once oxygen was depleted, the NADH/NAD^+^ couple responded to this, indicating that the mitochondria remained functional (Fig. 8).

**Fig. 9:**
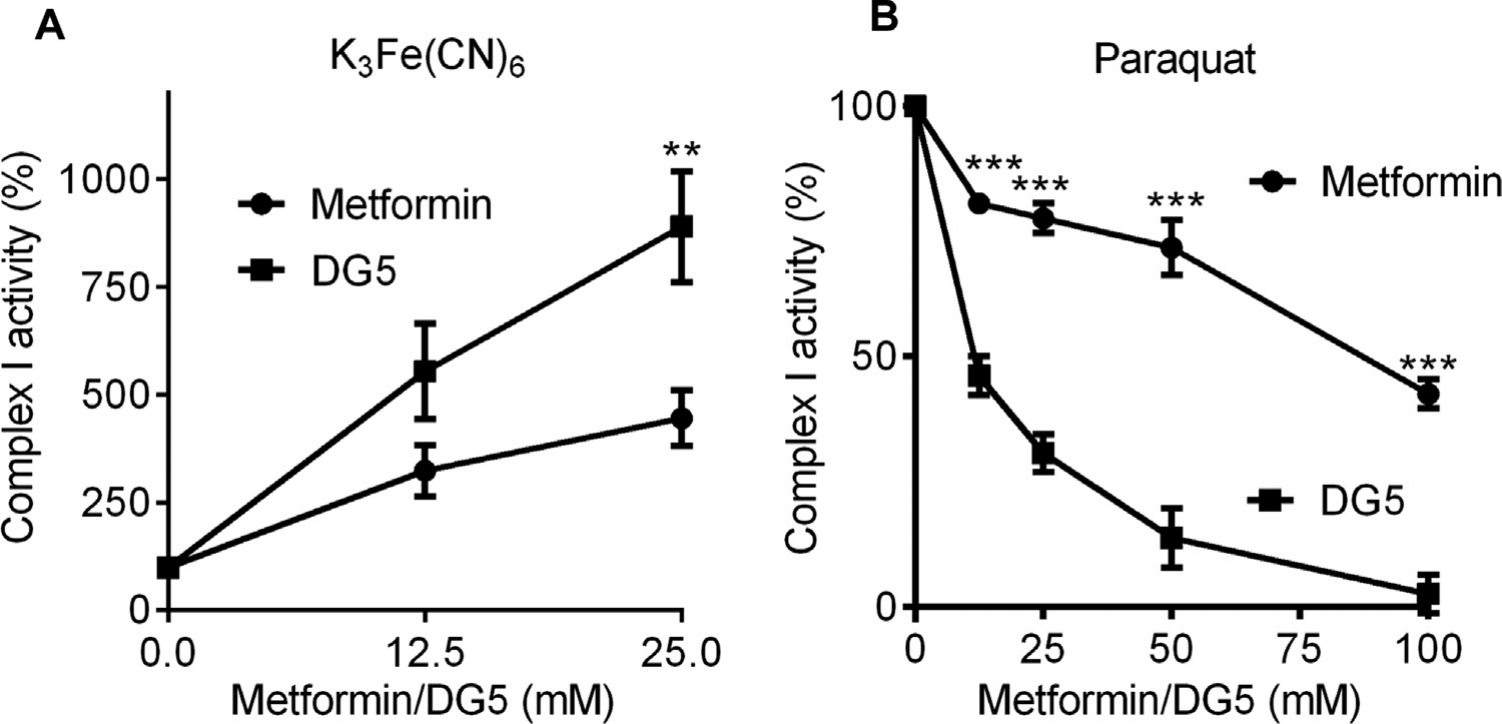
**Effect of metformin and DG5 on electron donation from complex I to hydrophilic electron acceptors.***(a,b)* The effect of dose responses of metformin and DG5 on reduction of K_3_Fe(CN)_6_ (a) and paraquat (b). A two-way ANOVA was carried out to determine differences between drug treatments. Each data point is composed of measurements of at least six individual wells.

In a final series of experiments, we compared metformin and DG5 on complex I electron transfer activity to artificial electron acceptors K_3_Fe(CN)_6_ and paraquat, as previous studies have shown that metformin has opposing effects on these, inhibiting NADH:paraquat electron transfer reactions, whilst stimulating NADH:ferricyanide electron transfer [30]. With metformin we observed similar effects to those published previously. The direction of the responses to DG5 was the same; however, there were important differences between the two drugs. The effects of metformin and DG5 on K_3_Fe(CN)_6_ had similar shaped dose-response curves, with >50% maximal activity achieved at 12.5 mM for both drugs (Fig. 9a). In contrast, particularly at low concentrations of drug, the dose-effect response of metformin on paraquat was much shallower than that observed with DG5 (Fig. 9b). Consequently the IC50 of metformin on electron donation to paraquat was almost an order of magnitude less potent than DG5 (metformin IC50 91 mM (95% CI 69–118); DG5 IC50 12 mM (95%CI 9–15)).

## 4 Discussion

### 4.1 Similarities and differences between cellular responses to biguanides and diguanides

In this study, we synthesized a series of diguanide compounds, to compare their action on cells with biguanides. Diguanides are metformin analogues where two guanide groups are separated by an alkyl chain of variable length. Extension of this chain from 4 to 10 carbons yields compounds with increasing hydrophobicity, and anti-hyperglycaemic efficacy. We investigated whether these compounds produced metformin-like effects on cell signalling, including elevation of AMPK phosphorylation and dephosphorylation of S6. We found that DG4 had little or no effect but DG5-DG10 had effects reminiscent of metformin and both DG5 and DG8 suppressed hepatocyte glucose production. Consistent with earlier in vivo data that these more hydrophobic diguanides are much more potent than metformin [11], we found that their effects occurred in the very low micromolar range, in striking contrast to metformin, where millimolar amounts of the drug are required for acute effects on signalling. In addition to the relationship between hydrophobicity and DG potency in unbranched diguanides, we found that branching of DGs, if it generated a hydrocarbon chain shorter than five carbons, resulted in compounds without activity on glucose production. These data indicate that the structure of the hydrocarbon chain also contributes to molecular recognition/access aspects of drug action, reminiscent of recent work on biguanide analogues [47].

The differences between biguanides and diguanides are consistent with the notion that metformin and diguanides employ different mechanisms once they access the mitochondria. The difference is unlikely to be due to the increased charge of diguanides, as monoalkylguanides, which possess a single charge like metformin, are even more potent inhibitors of mitochondria than diguanides [13]. We also measured the effects of biguanides and diguanides on gluconeogenic gene expression. At the longer time-point of these measurements, even DG4 reduced gluconeogenic gene expression much more potently than metformin. Phenformin was the most potent of the biguanides, within the range exhibited by diguanides, suggesting that phenformin but not metformin might be sufficiently hydrophobic to share a target with the diguanides.

### 4.2 Comparison of effects of biguanides and diguanides on mitochondrial responses

Using Seahorse microplate methodology, we found that biguanides and diguanides suppressed oxygen consumption rate (OCR) to a similar extent (Fig. 6) and with either drug type, the OCR following uncoupling with DNP was suppressed, consistent with previous data for metformin [2], [48] suggesting disruption of the electron transport chain. Differences between metformin and the other drugs were apparent however when we carried out more detailed investigations. First, we considered effects of the drugs on the NADH/NAD^+^ couple in SMPs and intact mitochondria, where the drugs are thought to accumulate. Previous results showed for example that biguanides inhibit NADH oxidation in SMPs at concentrations much higher than those required to induce respiratory inhibition in cells [14], [30]. The discrepancy in doses required is understood to owe at least in part to protracted accumulation of these drugs into the mitochondria, such that short term in vitro studies necessitate higher doses [30]. Expanded to include diguanides, we found that both phenformin and DG5 but not metformin inhibited NADH oxidation, suggesting that inhibition of complex I corresponds well with effects of these drugs on cell viability on mitochondrial dehydrogenase activity in cells. One possible reason for the differences between metformin and other drugs could be that responses to this drug are self-limiting, because metformin is understood to require active mitochondria in order to accumulate [14]; however in SMPs, the drugs have free access to the enzymes but even here metformin remains much less potent than phenformin and even a diguanide whose calculated hydrophobicity is less than metformin. The effects of phenformin and DG5 were also qualitatively different, with the inhibition by DG5 being instantaneous, whilst phenformin's effects took longer to develop and appeared similar to effects observed with 100 mM metformin added to SMPs, which were previously attributed to biguanides stabilizing ‘deactive’ forms of complex I [30], [49] that arise in the absence of NADH. This suggests that DGs may target active conformations of complex I, whereas biguanides selectively target the ‘deactive’ conformation, although mitochondrial swelling, which we have found is induced by phenformin (data not shown), provides an alternative explanation for the unusually slow development of effects on oxygen consumption we observed with this drug

Qualitative and quantitative differences were also observed in intact mitochondria. Despite a substantial reduction of the NADH/NAD+ couple by DG8 addition, oxygen consumption was only modestly inhibited. NADH is the only electron donor in this setting and so these results are difficult to interpret. Once oxygen was depleted, the NADH/NAD^+^ couple did not respond to this either. Taken together with the data in SMPs, this suggests that hydrophobic guanide-containing compounds but not metformin may to varying degrees trigger mitochondrial swelling and mitochondrial pore permeability transition. Similar slow inhibition of oxygen consumption of intact mitochondria by DG10 was described previously [13]. Metformin also reduced oxygen consumption but in striking contrast to DG8, the mitochondria did not deteriorate with metformin and the drug acutely oxidised the NADH/NAD^+^ couple (Fig. 8). Reduction of NADH/NAD+ following oxygen depletion suggests that substrate transport, which is mediated mainly by malate aspartate shuttle components in isolated mitochondria, is still functional even in the presence of high concentrations of metformin.

In recent in vitro studies it was shown that besides inhibiting NADH: hexaammineruthenium(III) and NADH:paraquat electron transfer reactions, metformin-dependent loosening of nucleotide binding from the reduced flavin stimulates NADH:ferricyanide and shunting of electrons from NADH directly to O_2_ with production of reactive oxygen species including superoxide [30], [50]. In addition to the differences we observed in intact mitochondria, we found that at modest doses, DG5 was a much more potent inhibitor than metformin of NADH:paraquat electron transfer, while both drugs exhibited a similar dose response curve of stimulation for NADH:ferricyanide electron transfer. The physiological significance of the differences in electron transfer to these hydrophilic electron acceptors is uncertain. Oxidation of NADH/NAD^+^ by metformin in intact mitochondria might suggest that stimulation of complex I electron transfer to generate reactive oxygen species is more significant for this drug, with complex I inhibition the more important effect for diguanides, with phenformin potentially exploiting both mechanisms. This suggests metformin but not diguanides may selectively divert NADH electron transfer to non-energy-transducing reactions, which could in principle explain some of the suppression of weight gain associated with use of the drug. Recent mutational analysis of bacterial complex I confirms that NADH oxidation and proton transfer coupling ratios are modifiable [51]. Control experiments suggest that the concentrations of metformin used do not cause mitochondrial swelling (data not shown) and in addition, similar differences on the NADH/NAD^+^ couple were obtained previously comparing phenformin with octylguanidine [52]. Taken together, these results suggest metformin, phenformin and diguanides each modulate mitochondrial oxidation of complex I substrates by distinct mechanisms.

## 5 Conclusion

A variety of guanide-containing drugs inhibit the mitochondria. We found that even a single guanide group attached to a lipophilic tail is a viable pharmacophore for these effects. We present additional evidence however that metformin does not produce the mitochondrial damage observed with more hydrophobic guanide-containing drugs, inhibiting the mitochondria by a distinctive mechanism resulting in oxidation of the NADH/NAD+ couple. Our results strongly suggest that metformin, but none of the other guanide-containing drugs tested, inhibits energy transduction by selectively suppressing efficient coupling of the redox and proton transfer domains of complex I. Further study of these differences might account for the clinical observations that hydrophobic alkylguanides and diguanides are toxic [12], hydrophobic biguanides such as buformin and phenformin are readily capable of inducing lactic acidosis [53], whilst metformin does not very readily exhibit either of these properties [54] but does suppress weight gain [29].

## Conflict of interest

No potential conflicts of interest relevant to this article were reported.
