# Supplementary material for: Metformin selectively targets redox control of complex I energy transduction
Source: Redox Biol. 2017 Aug 26;14:187–97. doi: 10.1016/j.redox.2017.08.018 (PMC5609876; doi:10.1016/j.redox.2017.08.018)
Supplement: Supplementary file 1 — Supplementary material [file mmc1.docx]

**Supplementary Materials**

**Supplementary Method**

**Purity Assessment by liquid chromatography mass spectrometry (LC-MS)**

The purity of the synthesised DG compounds was assessed using an adjustment of a previously published method for ion-pairing high performance liquid chromatography-mass spectrometry (HPLC-MS) [1]. Samples were analysed on a LCQ DECA Ion Trap mass spectrometer (Thermo Scientific Ltd, Hemel Hempstead, UK) attached to an HPLC system consisting of an Accella 600 quaternary pump and Acella photodiode array PDA detector (PDAD) and autosampler. The compounds were separated on a Synergi-Hydro-RP C18 column (Phenomenex Ltd, Macclesfield, UK; 150 x 2mm) using an ion pairing method. Samples (20 ml) were injected onto a gradient of solvent A (ultra-pure water containing 1mM heptafluorobutyric acid (HFBA) and solvent B (acetonitrile and 1mM HFBA). This gradient changed from 98%/2% combination of A/B to 40%/60% A/B over 30 mins. Then the column was washed with 100 % B and re-equilibrated for the next sample.

The separation phase (0-32 min) was analysed in the mass spectrometer using an electrospray ionization interface in positive-ion mode with two scan events: full-scan analysis followed by data-dependent MS^2^ of the most intense ions (normalised collision energies of 45% arbitrary units) in wideband activation mode.

**Reference**

**1.** Sanchez-Lopez J, Camanes G, Flors V, Vincent C, Pastor V, Vicedo B, Garcia-Agustin P et al, Underivatized polyamine analysis in plant samples by ion pair LC coupled with electrospray tandem mass spectrometry, Plant Physiology and Biochemistry, 2009, 47, 592-98

**Supplementary Figure Legends**

**Figure 1. Quantitation of blotting experiments in primary hepatocytes using commercially available analogues of metformin.**

Densitometry was carried out to quantify data obtained in western blots of pACC (a), pAMPK (b) and pS6 (c). Bars significantly different from the respective control treatment are shown, ***p<0.001. **p<0.01, *p<0.05. N=3

**Figure 2. Purity assessment of additional DG compounds**

*(a-d)* MS and MS^2^ spectra of DG5, DG7, DG8 and DG8N. All compounds had a signal at +214 M+H^+^ due to HFBA adducts. Major neutral losses are -42 = cyanamide (CH_2_N_2_), -59 = guanidine (CN_3_H_5_) and -17 = NH_3_. It is noticeable that DG8 and DG8N have completely different MS^2^ fragmentation, which confirms their different structure. The major loss of 116 in DG8N suggests that the diguanide breaks at the central N.

**Figure 3 & 4. Hydrophobicity maps of selected structures used in this study**

Hydrophobicity maps are presented of DG8N (2a), DG8 (2b), metformin (3a) and phenformin (3b)

**Figure 5. Quantitation of blotting experiments in primary hepatocytes using DG5, DG8 and DG8N.**

Densitometry was carried out to quantify data obtained in western blots of pACC (a), pAMPK (b) and IκB (c). Bars significantly different from the respective control treatment (+/- TNFα) are shown, ***p<0.001. **p<0.01, p<0.05. N=3

**Figure 6. Purity assessment of branched DG compounds**

*(a)* MS and *(b)* MS^2^ of branched compounds. The red m/z signals are from HFBA adducts of the products (+214). The levels of this adduct may reflect the structure of the DG.

**Supplementary Fig. 1a.**

**

**

**Supplementary Fig. 1b.**

**

**

**Supplementary Fig. 1c.**

**

**

**Supplementary Fig. 2a. MS and MS^2^ spectra of DG5**

FSD = 1.37e7

100

150

200

250

300

350

400

450

500

0

10

20

30

40

50

60

70

80

90

100

Relative Abundance

**187.09**

400.75

188.09

170.08

128.15

383.16

431.16

FSD = 1.10e6

100

120

140

160

180

200

220

240

***m/z***

0

10

20

30

40

50

60

70

80

90

100

Relative Abundance

145.04

128.09

187.01

144.41

110.97

153.06

167.88

**Supplementary Fig. 2b. MS and MS^2^ spectra of DG7**

FSD = 2.32e7

100

150

200

250

300

350

400

450

500

0

10

20

30

40

50

60

70

80

90

100

Relative Abundance

**215.15**

428.82

216.16

108.17

198.17

128.18

FSD = 5.22e6

100

120

140

160

180

200

220

240

***m/z***

0

10

20

30

40

50

60

70

80

90

100

Relative Abundance

173.08

156.06

181.10

138.99

131.03

198.08

215.03

114.10

164.22

155.25

**Supplementary Fig. 2c. MS and MS^2^ spectra of DG8**

FSD = 2.64e7

100

150

200

250

300

350

400

450

500

0

10

20

30

40

50

60

70

80

90

100

Relative Abundance

**229.18**

442.84

115.21

230.19

443.91

135.12

212.19

187.20

FSD = 5.79e6

100

120

140

160

180

200

220

240

***m/z***

0

10

20

30

40

50

60

70

80

90

100

Relative Abundance

187.12

170.11

195.10

153.12

145.09

212.12

229.12

128.08

110.83

**Supplementary Fig. 2d. MS and MS^2^ spectra of DG8N**

FSD = 1.03e7

100

150

200

250

300

350

400

450

500

0

10

20

30

40

50

60

70

80

90

100

Relative Abundance

**230.10**

443.84

231.17

115.62

213.23

135.71

171.13

FSD = 3.18E6

100

120

140

160

180

200

220

240

***m/z***

0

10

20

30

40

50

60

70

80

90

100

Relative Abundance

114.14

196.10

188.13

171.14

213.22

154.28

129.01

155.14

**Supplementary Fig. 3a**

**DG8N**

**
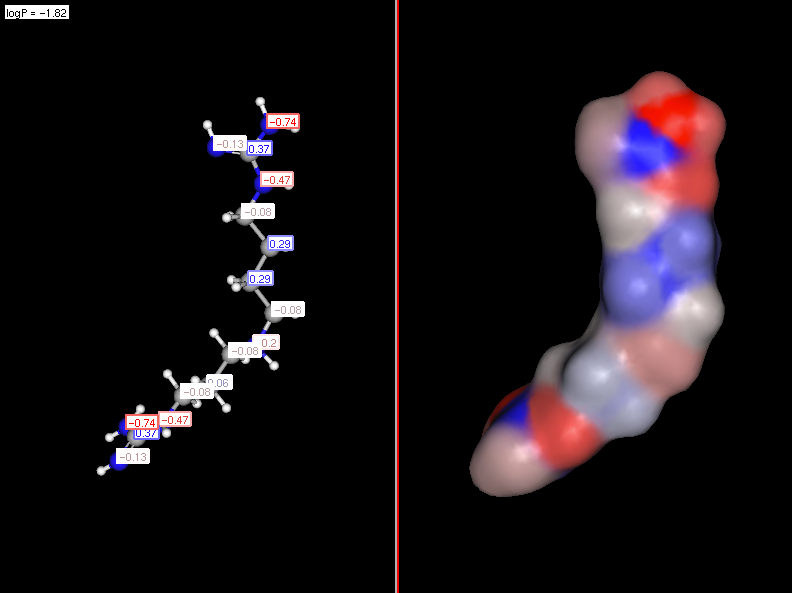
**

**Supplementary Fig. 3b**

**DG8**

**
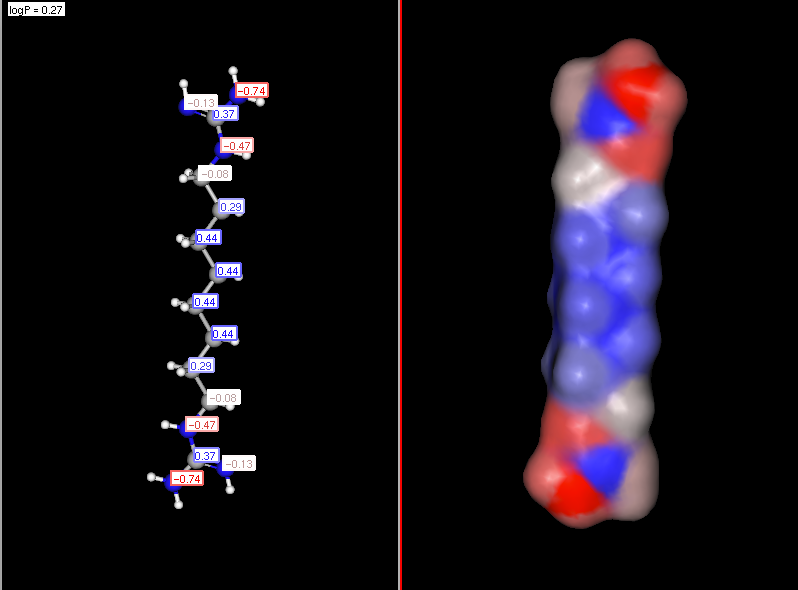
**

**Supplementary Fig. 4a**

**Metformin**

**
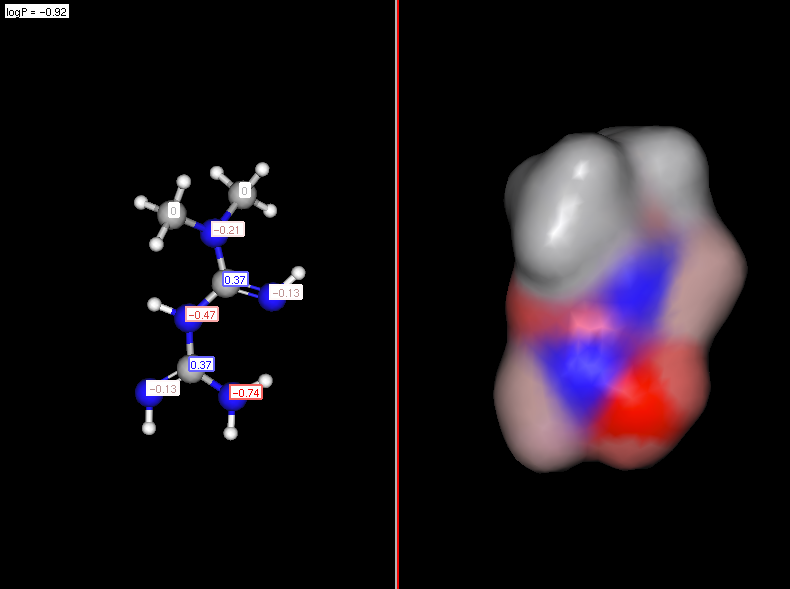
**

**Supplementary Fig. 4b**

**Phenformin**

**
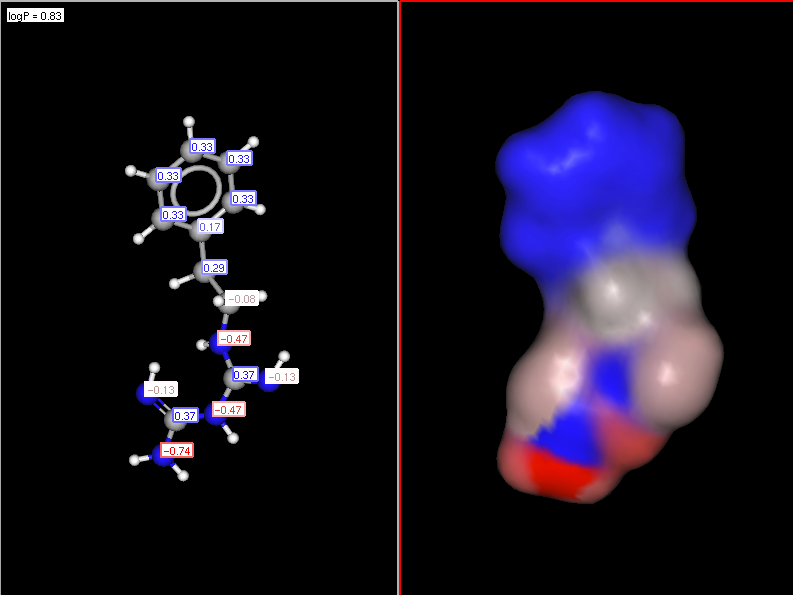
**

**Supplementary Fig. 5a**

**

**

**Supplementary Fig. 5b

**

**Supplementary Fig. 5c

**

**Supplementary Fig. 6**

100

200

300

400

500

*m/z*

0

20

40

60

80

100

0

20

40

60

80

100

Relative Abundance

0

20

40

60

80

100

201.15

202.16

414.83

187.06

188.07

400.81

187.11

400.86

188.13

5.24e8

**2-Methyl DG5**

5.81e8

6.27e8

**A** M+H mass spectra

**1-ethyl DG5**

**isomer**

**2,2 dimethyl DG5**

**isomer**

**B** MS^2^ fragmentation

100

200

*m/z*

0

20

40

60

80

100

0

20

40

60

80

100

Relative Abundance

0

20

40

60

80

100

159.01

142.13

59.94

99.99

167.13

128.05

153.04

170.05

145.02

128.03

59.84

85.96

153.02

9.24e6

1.01e7

1.98e7

145.02

**1-ethyl DG5**

**isomer**

**2-Methyl DG5**

**2,2 dimethyl DG5**

**isomer**
